# Data on risk preferences and risk literacy for a sample of German agricultural sciences students

**DOI:** 10.1016/j.dib.2018.04.016

**Published:** 2018-04-10

**Authors:** Manuela Meraner, Oliver Musshoff, Robert Finger

**Affiliations:** a Agricultural Economics and Policy Group, ETH Zurich, Sonneggstrasse 33, 8092 Zürich, Switzerland; b Production Economics Group, Institute for Food and Resource Economics, University of Bonn, Meckenheimer Allee 174, 53115 Bonn, Germany; c Department of Agricultural Economics and Rural Development, University of Göttingen, Platz der Göttinger Sieben 5, 37073 Göttingen, Germany

## Abstract

The data presented here contains information on risk preferences, risk literacy and personal characteristics collected from 244 German agricultural sciences students in an online survey in 2015. Two different risk preference elicitation methods have been used. First, we used an iterative multiple price list (iMPL). Second, a simple self-assessment of risk preferences was used. Moreover, we used two different frames of the iMPL (general and context specific). Inconsistent behavior within the iMPL has been documented. Finally, the dataset includes information on the participants’ risk literacy (using the Berlin Numeracy test), gender, optimism, involvement with agriculture age and mothers’ education. The data is related to the paper: Meraner M, Musshoff O, Finger R. Using involvement to reduce inconsistencies in risk preference elicitation. Journal of Behavioral and Experimental Economics. 2018 73:22–33.

**Specifications Table**

**Table:** 

Subject area	*Experimental Economics, Behavioral Economics*
More specific subject area	*Risk preference elicitation*
Type of data	*CSV File*
How data was acquired	*Online survey*
Data format	*Raw data, partially analyzed*
Experimental factors	*No pretreatment of sample*
Experimental features	*Very brief experimental description*
Data source location	*Bonn and Soest, North-Rhine Westphalia, Germany*
Data accessibility	*With this article.*

**Value of the data**

• The data allows for comparison of risk preferences with other case studies in meta-analyses.
• The data allows to study within- and between-method inconsistencies in risk preference elicitation.
• The data allows for comparison of risk literacy as measured with the Berlin Numeracy test.

## 1 Data

The data includes results from an online survey sample of 244 German agricultural students. It contains results of two different risk preference elicitation methods: an iterative multiple price list (iMPL) following Harrison et al. [1] and self-assessment of risk preferences following Dohmen et al. [2]. Two different frames of the iMPL have been applied and inconsistent behavior within the iMPL has been documented. Finally, the dataset includes information on the participants risk literacy (using the Berlin Numeracy test), gender, optimism, involvement with agriculture, age and mothers’ education.

## 2 Experimental design, materials and methods

An online survey was conducted at the two largest agricultural faculties in North-Rhine Westphalia, i.e. the agricultural faculty of the University of Bonn and the South Westphalian University of Applied Sciences (located in Soest). All agricultural sciences students in both universities were invited to participate in two separate but identical online surveys conducted in January and March 2015, respectively. We obtained 156 complete questionnaires from Bonn University and 96 from the South Westphalian University of Applied Sciences leading to 252 complete questionnaires (representing a response rate of 15%). After the data cleansing process 244 surveys remained. More specifically, we removed participants who were not enrolled in agricultural studies at these two universities and we excluded non-German students to eliminate biases due to different educational backgrounds and cultural differences we are not accounting for in our survey.

The experiment was conducted in two parts. Part I consisted of two risk preference elicitation tasks and part II consisted of a questionnaire collecting subjects’ socio-demographic characteristics. More specifically, we collected information on age, sex, optimism, mothers’ highest educational degree and risk literacy. For the latter, we used the Berlin Numeracy test described in Cokely et al. [3]. Additionally, we included in this section specific characteristics to measure the students’ involvement with agriculture (i.e. by asking whether students grew up on a farm holding, parents are farmers, planned succession of a farm, type and length of specific agricultural education). From this information, we derived an indicator that represents the students’ general involvement in agriculture. Furthermore, with the software used to program our survey (Lime Survey) we were able to measure the time each participant spent on each part of the questionnaire. The time spent in both risk elicitation tasks is presented in this dataset. The data was collected anonymously and voluntary, all participants where made aware of the anonymized data processing procedure after the end of the data collection.

To elicit risk preferences we use two methods dominant in the literature: a self-assessment of general risk preferences, and an iterative Multiple Price List (iMPL), an extension of the Multiple Price List (MPL) developed Harrison et al. [1]. It elicits risk preferences, resulting in a more refined description of the subjects’ risk preferences compared to the standard MPL. The standard MPL as introduced by Holt and Laury [4] is structured as follows: the table has 10 rows and two columns; in each row the subjects face two gambling choices A and B (see Meraner et al. [6], Table 1). In the iMPL the subjects are presented a second table with probabilities altering in-between the switching point of the first basic MPL. Assume, for example, that a subject switches in the first table in the third row from A to B (note that this is the same as to say the subject has chosen two safe choices). Note that the iMPL was incentivized, i.e. participants received payouts according to their choices in the lottery (see Meraner et al., 2018 and Appendix A for details).

**Table 1:** Variable description.

**Variable**	**Variable Name**	**riable Definition**
iMPL1	Decision_1(1=Safe)	Decision in first row of iMPL A = 1
iMPL2	Decision_2	Decision in second row of iMPL A = 1
iMPL3	Decision_3	Decision in third row of iMPL A = 1
iMPL4	Decision_4	Decision in fourth row of iMPL A = 1
iMPL5	Decision_5	Decision in fifth row of iMPL A = 1
iMPL6	Decision_6	Decision in sixth row of iMPL A = 1
iMPL7	Decision_7	Decision in seventh row of iMPL A = 1
iMPL8	Decision_8	Decision in eight row of iMPL A = 1
iMPL9	Decision_9	Decision in ninth row of iMPL A = 1
iMPL10	Decision_10	Decision in tenth row of iMPL A = 1
iMPL11	Decision_11	Decision in eleventh row of iMPL A = 1
iMPL12	Decision_12	Decision in twelfth row of iMPL A = 1
iMPL13	Decision_13	Decision in thirteenth row of iMPL A = 1
iMPL14	Decision_14	Decision in fourteenth row of iMPL A = 1
iMPL15	Decision_15	Decision in fifteenth row of iMPL A = 1
iMPL16	Decision_16	Decision in sixteenth row of iMPL A = 1
iMPL17	Decision_17	Decision in seventeenth row of iMPL A = 1
iMPL18	Decision_18	Decision in eighteenth row of iMPL A = 1
iMPL19	Decision_19	Decision in nineteenth row of iMPL A = 1
iMPL20	Decision_20	Decision in twentieth row of iMPL A = 1
SumSafe	SumSafe	Sum of safe decisions in iMPL (=A in first ten questions)
Risk preference from CRRA interval mid-point	CRRA interval mid-point	Midpoint of constant relative risk aversion interval of the first switch from Option A to B in iMPL, assuming a power utility function *U*(*x*) = (1−*r*)-1 × 1−*r*.
Risk preference from self-assessment	Self-assessment	0 if very risk averse; …; 10 if very risk loving
Frame iMPL	dummy_agric	1 if agricultural decision frame of iMPL, 0 if general lottery frame of iMPL
Inconsistent behavior	dummy_inc	1 if inconsistent behaviour in iMPL
Risk literacy score	RL_score	1 = poor numerical reasoning; 2 = rather poor numerical reasoning; 3 = good numerical reasoning; 4 = very good numerical reasoning
Gender	Gender	1 if female
Optimism	Opt	Difference of life satisfaction in a year and life satisfaction today (both measured on a scale from 0 to 10)
*Rural origin*	inv_Rural_origin	0.5 if area of growing up has less than 20,000 inhabitants
*Growing up on farm holding*	inv_grew up on farm	1 if grew up on a farm
*Parents are farmers*	inv_parentas_farmers	1 if parents are farmers
*Succession of farm holding intended*	inv_successor	0.5 if probably no succession is intended; 1 if probably succession is intended; 2 if succession is intended
*Agricultural Internship*	inv_intrnship	0.5 if internship time is less or equal to 6 months; 1 if internship time is more than 6 months
*Vocational training*	inv_vocational_training	1 if agriculture specific vocational training obtained
*Agricultural school*	inv_agricultural_school	1 if three year agricultural school degree
*Master exam*	inv_agric_master	1 if five year agricultural school degree (master)
*Higher agricultural education*	inv_higher_agric_educ	1 if higher agricultural education obtained
Involvement score	Involvement_score	Sum of involvement factors described above
Age	Age	Years
Education mother	education_mother	Mothers highest education according to the German schooling system: = 1if no degree obtained; = 2 if lower secondary qualification, = 3 if higher secondary qualification, = 4 if advanced technical certificate, = 5 if high school degree, = 6 if applied university degree, 7 = if university degree, = 8 if PhD degree
Time iMPL	time_iMPL	Time spent on iMPL in minutes
Time self-assessment	time_self-assessment	Time spent on self-assessment of risk preferences in minutes
Risk preference classified depending on task involvement	CRRA_l_highse	CRRA interval mid-point with lottery frame and high task involvement
	CRRA_l_lowse	CRRA interval mid-point with lottery frame and low task involvement
	SA_l_highse	Self-assessment score with lottery frame and high task involvement
	SA_l_lowse	Self-assessment score with lottery frame and low task involvement
	CRRA_i_highse	CRRA interval mid-point with agricultural frame and high task involvement
	CRRA_i_lowse	CRRA interval mid-point with agricultural frame and low task involvement
	SA_i_highse	Self-assessment score with agricultural frame and high task involvement
	SA_i_lowse	Self-assessment score with agricultural frame and low task involvement

To analyze the data obtained in terms of coefficients of risk aversion we assume under expected utility theory the subjects’ utility function to have the following constant relative risk aversion (CRRA) form: *U*(*x*) = (1−*r*)^−^^1^ × ^1−*r*^, where *x* is the lottery price (investment return) and *r ≠ 1* the parameter of risk aversion to be estimated. With this functional form, *r* = 0 denotes risk-neutral behavior, *r* > 0 denotes risk aversion, and *r* < 0 denotes risk-loving behavior. By minimizing the difference in expected utilities obtained from option A and option B we can calibrate the open CRRA interval.

We include two different decision frames in our experiment, i.e. two different wordings that change the contextual setting of the iMPL. More specifically, we included next to general lottery description an agriculture specific wording (see Meraner et al., 2018). Additionally, we randomly changed the order of the two risk preference elicitation methods (i.e. self-assessment and iMPL). By using a random design assigning each participant only one frame, we aim to control for potential biases arising from the sequence of tasks. Further descriptions of each variable included can be found in Table 1. The instructions to the risk elicitation tasks presented to the subjects are available in Appendix A.

Based on the decisions in the iMPL task, we measured inconsistencies in this risk elicitation task. More specifically, different ways of inconsistent behavior are accounted for: i) inconsistent response behavior is revealed if more than one switching point between option A and B is observed; ii) inconsistent behavior is indicated by “backwards” choices, i.e. switching in the other direction from option B in the first row to option A in the following rows [4], [5] and iii) as the last set of choices is commonly a control question with option B clearly dominating option A, a subject choosing A in all 10 rows is also thought of behaving inconsistent, because in the last row option B results with certainty in a higher payoff than option A (see also Meraner et al. [6], Table 1, for an example). Note that in the iMPL there is a possibility of inconsistent behavior either in the first or in the second table. Both cases are in the following treated as within-method inconsistencies.
