# Supplementary material for: Data on risk preferences and risk literacy for a sample of German agricultural sciences students
Source: Data Brief. 2018 Apr 10;18:1267–71. doi: 10.1016/j.dib.2018.04.016 (PMC5996949; doi:10.1016/j.dib.2018.04.016)
Supplement: Supplementary file 2 — Supplementary material [file mmc2.docx]

## Appendix B

#### First screen

Welcome to the experiment!

In the following we will offer you various situations and options to choose from. We would like to get to know something about your behavior in different situations/scenarios. There are no 'right' or 'wrong' decisions!

For all participants there is a chance to be drawn at random for a win of 87 €. We will inform you about your payoff via e-mail. The payoff of the win will be carried out immediately after the evaluation of the experiment.

The experiment will take approx. 20 minutes. Of course, your data will be treated confidentially and the data will be evaluated anonymously. For further inquiries please contact: m.meraner@ilr.uni-bonn.de.

#### Second screen

Please read carefully through the following description of your chances to win:

**What can you win?** The payoff for each participant can amount up to 87 € (first part: 77 € + second part: 10 €).

**How can you win?** In the first part of the experiment you will be presented a table with ten rows. For each row you will have to choose between option A and option B. The decision (row), that will be relevant for your payoffs, will be determined by the first draw out of a lottery with ten balls. In the case that row 4 will be identified, you were asked to choose between option A (40% probability/chance 40.000 € und 60% probability/chance 32.000 €) and option B (40% probability/chance 77.000 € and 60% probability/chance 2.000 €). Your win will be multiplied by the factor 1/1.000.

If we assume that you have chosen option B in the randomly selected row 4. In the second draw, the numbers 1 to 4 (= 40% chance) lead to a payoff of 77 €, the numbers 5-10 (= 60% chance) result in a payoff of 2 €. In the second part we will ask you to solve some arithmetic questions. For the correct answer to these tasks, the participants, who were selected as winners, receive additionally 10 €.

**Who can win?** 10% of all participants will be drawn at random to receive the payment.

#### Third screen: control question

To make sure, that you understood the method of payment for the reward of your participation, please answer the following question:

Please assume that you were drawn randomly as one of the winners. In the first draw, which serves to identify the row, that will be relevant for the payment, 4 out of 10 was drawn. This means that the decision row 4 will be relevant for your payment. Assume furthermore, that you have chosen option A in the relevant decision (marked with the blue dot in the table on the right).

Example: (see Figure 1)

The second draw results in number 7. What is the amount of your payoff?

(1.000 € in the lottery = 1 € payoff).

(Right answer not shown to participants: 32)

**
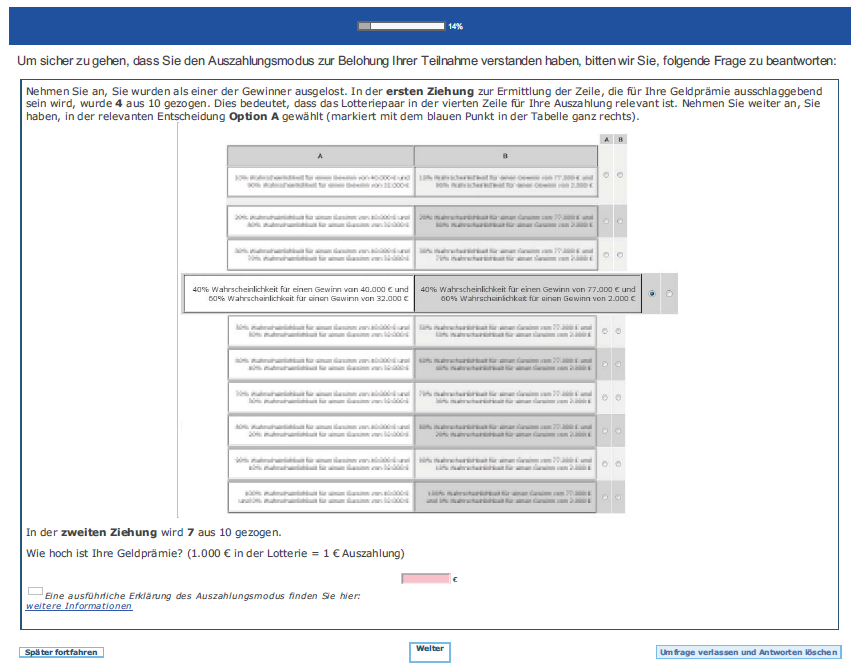
**

**Fig 1B:** Third screen: control question

Only one of the following two screens is shown:

#### Fourth screen A: General lottery task

In each row of the following table you can choose between two lotteries (A and B). With certain chances/ probabilities you get for lottery A a payoff of 40.000 € or 32.000 € and for lottery B a payoff of 77.000 € or 2.000 €. Please decide between lottery A and B for every row of the table.

#### Fourth screen B: Agricultural decision task

Assume that after successfully completing your studies you are offered to make an agricultural investment. Here you will get with different associated probabilities for investment A a return of 40,000 € or 32,000 € and for investment B a return of 77,000 € or 2,000 €. You can choose in the following table in each row between the two investment options (A or B). Please decide between investment A and B for every row of the table.


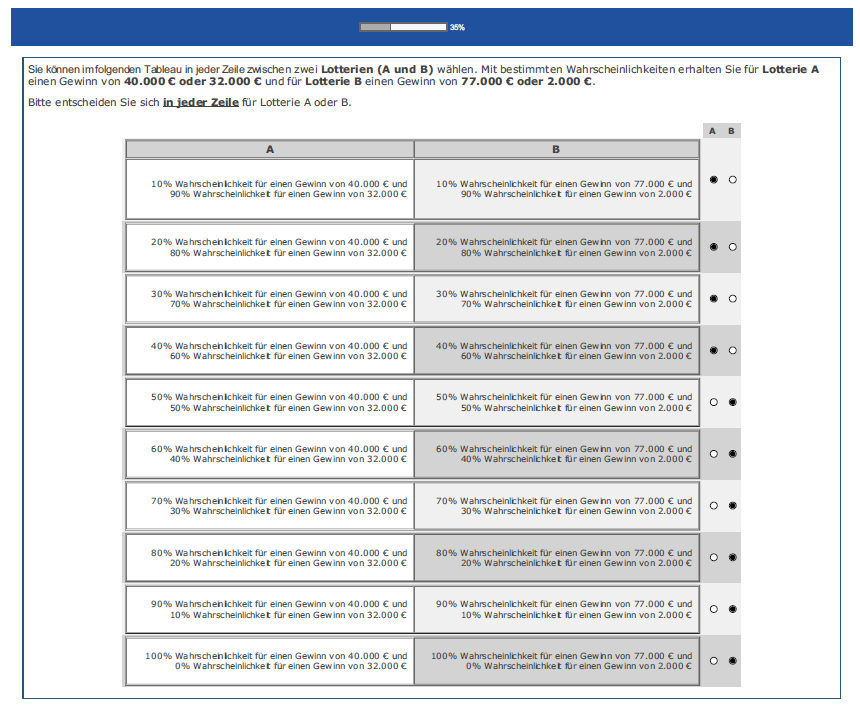


**Fig 2B:** Fourth screen: General lottery task

#### Fifth screen: Self-assessment

How do you see yourself: are you generally a person who is fully prepared to take risks or do you try to avoid taking risks? Please tick a box on the scale, where the value 0 means: 'not at all willing to take risks' and the value 10 means: 'very willing to take risks'.


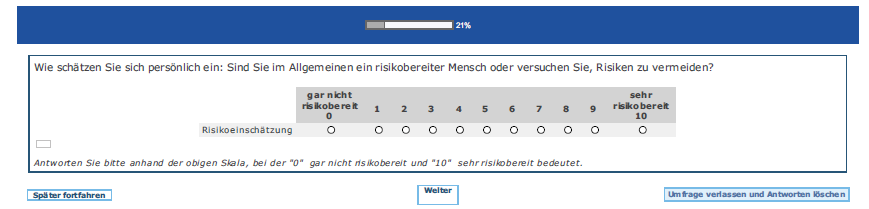


**Fig 3B:** Fifth screen: Self-assessment task
